# Effects of Roller Milling Parameters on Wheat-Flour Damaged Starch: A Comprehensive Passage Analysis and Response-Surface Methodology Optimization

**DOI:** 10.3390/foods13213386

**Published:** 2024-10-24

**Authors:** Nemanja Bojanić, Dušan Rakić, Aleksandar Fišteš

**Affiliations:** Faculty of Technology, University of Novi Sad, Bul. cara Lazara 1, 21000 Novi Sad, Serbia; drakic@tf.uns.ac.rs (D.R.); fistes@uns.ac.rs (A.F.)

**Keywords:** wheat, industrial flour milling, flour stream analysis, starch damage, roll settings

## Abstract

Damaged starch typically arises from mechanical damage caused by the action of the roller mills during the wheat flour milling process. The content of resulting damaged starch in the flour significantly influences its characteristics, emphasizing the importance of understanding and controlling the formation of damaged starch for the production of specialized flours. A detailed passage analysis from three different commercial mills revealed that starch damage control is primarily achievable at front passages of the sizing and reduction system, which generate the majority of the flour release in the mill. Also, it revealed that damaged starch content increases progressively from the initial to the final passages during milling in the break, sizing and reduction system. To investigate the effects of milling parameters on damaged starch, flour yield, and energy consumption, a three-level and three-variable Box–Behnken experimental design with response surface methodology was applied. As independent variables roll gap (0.05–0.35 mm), feed rate (0.15–0.35 kg/cm min), and fast roll speed (400–800 rpm) were employed. The obtained models were utilized to optimize milling conditions for producing flours with special characteristics.

## 1. Introduction

Damaged starch refers to the alteration in the structure of starch molecules, impacting their functionality and properties [1]. Structural damage of starch can result from various factors like storage conditions, processing or cooking [2]. In the food industry, understanding and controlling damaged starch levels are important for achieving the desired product attributes, since they affect food texture, viscosity, and overall product quality [3]. For example, damaged starch exhibits higher water absorption and enzymatic hydrolysis rates, making it easily fermentable by yeasts. A moderate level of starch damage can enhance dough quality in bread making, but excessive damage can lead to sticky dough [4]. because of its importance, various studies have been employed recently, to investigate a wide array of functional properties, such as gelatinization, pasting properties, water hydration properties, and enzymatic digestibility of damaged starch [5,6,7,8] as well as starch-based food systems [9,10,11]. The optimal starch-damage content depends on the specific type of baked product. Approximate levels of damaged starch, measured in UCD units, can range from 13.7 to 16.4 for cookies, 15 to 17.5 for noodles, and up to 19 to 23 for pan bread [12].

In the wheat flour milling industry, the production of damaged starch is mainly influenced by three factors: the type of wheat, the wheat conditioning process, and, primarily, by the milling process [13,14]. The flour milling process involves the multiple and consecutive processes of wheat and intermediate stocks grinding and sieving, through a number of so-called grinding passages, whereas as a milling machine is predominantly used the roller mill [15]. During the milling process, friction, heat, and mechanical forces are exerted on the milled material and thus can lead to the breakage and alteration of starch granules [4]. The nature and intensity of deformation forces acting on the milled material are determined by the milling parameters [16], i.e., the set of roll parameters such as roll gap, differential, roll speed, etc. Therefore, controlling the influence of these parameters is necessary to achieve a satisfactory level of damaged starch in flour. Additionally, milling parameters affect flour yield, energy consumption, and other indicators of milling efficiency. Thus, the primary task in wheat flour production is to optimize milling parameters to achieve a satisfactory product while minimizing costs.

Various studies on the topic have involved tracking the generation of damaged starch due to different types of milling, as well as investigating the impact of milling parameters on the content of damaged starch in the final product [1,17,18,19]. Numerous studies have extensively explored the physicochemical properties, rheological behavior, and bread-baking performance of individual mill streams, with a specific focus on damaged starch generation [20,21,22]. Scanlon et al. [17] reported that the increase in the differential or narrowing of the roll gap increased the damaged starch content of the flour. However, the roll gap setting was shown not to influence the degree of starch damage in the fine fraction of the flour. Also, Ghodke et al. [23], implemented response surface methodology in order to explore how stone mill aperture, feed rate, and moisture of wheat grain affect damaged starch content in whole-wheat flour, as well as dough stickiness and chapatti (Indian unleavened flat bread) quality. However, to the best of the authors’ knowledge, no study has yet been conducted that involved a detailed passage analysis of milling flows from multiple commercial mills followed by the optimization of individual, damaged starch most influential passage, using RSM methodology. Additionally, the obtained models were used for the optimization of milling parameters and in accordance with the targeted content of damaged starch, to produce specific types of baked products.

Therefore, in this study, the mill flows of three commercial mills were observed, and a passage analysis was employed. Flour yields, ash content, and damaged starch content in the passage flours were analyzed. An assessment was made to determine how the generation of the damaged starch is distributed through milling phases and passages. Due to the fact that the highest proportion of damaged starch is generated in the reduction system of the flour milling process, the impact of milling parameters on damaged starch and other milling efficiency indicators was investigated in the second phase of the study. Smooth rolls commonly used in industrial milling were employed, along with other milling parameters aligned with industrial practices. For that purpose, the response surface methodology was used and the Box–Behnken experimental design was utilized. The input parameters considered were roll gap, feed rate, and roll speed, while as the responses, the content of damaged starch in the flour, flour yield, and energy consumption were observed. The obtained models were further analyzed to explore the potential for producing wheat flour suitable for various types of end products under optimal conditions.

## 2. Materials and Methods

### 2.1. First Stage

#### 2.1.1. Samples

Samples of intermediate flour streams were systematically collected from three commercial wheat flour mills in Serbia, denoted as mill A, B, and C, with the capacities of 250, 125, and 180 t/day, respectively. Each mill generously contributed samples of individual flour streams from all milling stages, accompanied by detailed information regarding the yield of each passage flour. However, information on the mill mixtures was not provided by the mills.

#### 2.1.2. Sample Analysis

Sample analysis encompassed the determination of ash content, moisture content, particle size distribution and the degree of the damaged starch. Ash content and moisture content have been determined according to ICC standard methods [24,25], while particle size distribution was measured by the laser light scattering method using Mastersizer Scirocco 2000 (Malvern Instruments, Worcestershire, UK). The results obtained were presented through two dependent parameters: volume-weighted mean diameter, De Brouckere Mean Diameter, (VD) (µm) (D4.3) and volume median diameter D (0.5) (µm).

Parameters of damaged starch were determined using the Chopin iodometric method with the SD Matic device, which is designed to measure the starch damage based on the percent of iodine absorption (Ai%) of the flour, and is able to convert that result into UCDs (Chopin Dubois Units), AACC 76-31 and Farrand units [26,27]. The measurements were conducted in duplicate for all samples, and in triplicate for the samples where the difference between the first two measurements was higher than 1 UCD unit.

### 2.2. Second Stage

#### 2.2.1. Sample

The sample which represents purified middlings was acquired from a local industrial wheat flour mill by intercepting the stream leaving the purifier. This material is alternatively considered as fine semolina. The total mass of the sample was 50 kg. The sample was firstly characterized in terms of granulation (using Mastersizer Scirocco 2000), ash content [24] and moisture content [25]. Afterwards, the material was homogenized and then separated using the automatic sampler divider (Gompper–Maschinen KG, Tuttlingen, Germany) into 500 g batches which were used as the milling sample.

#### 2.2.2. Milling of Middlings

For each experiment, 500 ± 0.01 g of cleaned wheat middlings were milled on the laboratory roll stand Variostuhl, model C Ex 2 (Miag, Braunschweig, Germany), equipped with smooth rolls (100 mm length and 250 mm diameter) having a frosted finish. All operating parameters of the mill were varied at three levels, according to the experimental plan. Roll gap (0.05, 0.2 and 0.35 mm), feed rate (0.15, 0.25 and 0.35 kg/cm min) and fast roll speed (400, 600 and 800 rpm) were employed as independent variables. The differential was set to be 1.25, while the upper and lower levels for the input parameters were chosen based on the previous research associated with the investigated topic [22] and, as in the case of the differential, chosen to be near the ranges that are likely to occur in commercially milling. The responsive variables were R1: the iodine absorption Ai (%), R2: damaged starch content in UCD, R3: flour yield (%) and R4: energy consumption (wh/kg).

#### 2.2.3. Flour Analysis

Sieve analysis of the milled stock was performed on the Buhler laboratory sifter (gyratory in a horizontal plane), model MLU-300 (Uzwil, Switzerland). Every milled sample (every 17 batches of 500 g) were first divided using Gompper–Maschinen KG into 100 g batches, and those samples were used for sieving. Samples were sieved for 3 min in order to obtain flour (<150 µm). The stock held on a bottom collecting pan was weighed to 0.01 g using a Sartorius Precision balance (Sartorius AG, Göttingen, Germany), and flour yield was given relative to the mass of input material. Parameters of damaged starch in the thus-obtained flour fractions were determined using the Chopin iodometric method with the SD Matic device. For each measurement, samples of 1 g were used.

#### 2.2.4. Energy Consumption

An integral part of the laboratory roll stand is an instrument for measuring the power (kW) required for the roll stand to operate without and/or with material flow. This instrument was used to obtain data for calculation of the total energy consumption (E [wh/kg]) during the milling operation, according to the following equation:

(1) $$E=\frac{P_{w}}{M\cdot 3600}\cdot t$$

Equation (1) was used to calculate energy consumption in relation to the mass of the milled sample. Here, P_w_ (W) represents the power reading corresponding to the operation of the roll stand with the material flow. The time of the grinding run determined by the chronometer is denoted by t (s), while M (kg) stands for the weight of the native feed.

#### 2.2.5. Statistical Analysis

Experimental runs were performed according to the Box–Behnken experimental design (BBD), with three independent process parameters at three levels and with five replicates at the central point. This design reduces the number of runs from 27 (full factorial design) to 17, with sufficient information for testing of the lack of fit, since five central point were included. The three independent process parameters were A—roll gap, B—feed rate, and C—roll speed. Damaged starch parameters Ai (%) and UCD, flour yield (%), and energy consumption in relation to milled material (wh/kg) have been observed as responses R1–R4, respectively. The regression analysis was performed and model is described by the polynomial of second order:

(2) $$R=\beta_{0}+\beta_{1}A+\beta_{2}B+\beta_{3}C+\beta_{12}AB+\beta_{13}AC+\beta_{23}BC+\;\beta_{11}A^{2}+\beta_{22}B^{2}+\beta_{33}C^{2}$$

In following Equation (2), R is a measured response; β_0_ is an intercept; β_1_ to β_33_ are regression coefficients; and A, B, and C are the coded levels of input factors. The terms AB, AC, and BC represent interactions of input factors, while A^2^, B^2^, and C^2^ represent quadratic terms. Model adequacy checking is carried out by calculating the R^2^ and “Lack of Fit” (LoF) coefficients. The significance of input factors and their interactions in the observed model are determined by the statistical method of analysis of variance (ANOVA). Using 5% level of significance, a factor is considered as statistically significant if the *p* value is less than 0.05. The sum of squares obtained by ANOVA are used to calculate the corresponding contributions. The analysis was carried out using Design–Expert 11 [28].

## 3. Results and Discussion

The following research was conducted in two stages. Within the first stage, an assessment was made to determine how generation of the damaged starch is distributed through milling phases and passages. In the second stage, the impact of milling parameters on damaged starch and other milling efficiency indicators was investigated. Additionally, the obtained models were further analyzed to explore the potential for producing wheat flour suitable for various types of end products under optimal conditions.

### 3.1. First Stage

In Table 1, a comprehensive overview of three flour mills, detailing their respective grinding passages, the number of extracted flours from each passage, and the corresponding yield and starch damage of the resultant flours is presented. Further, in the Appendix A (Table A1, Table A2 and Table A3), a more intricate analysis of each mill’s passages is provided, offering detailed information on passage-specific characteristics.

The SD Matic device working principle is based on the measuring of iodine absorption levels and, in such cases, the results are independent of flour granulation. On the other hand, when expressing results in UCD units, the device incorporates particle size considerations in the calculation. Consequently, in cases where coarser granulation of flour is present, UCD values tend to be unrealistically low, occasionally even taking on negative values. In the case of Mill A, flour streams with negative or low values were observed in passages CL1, CL2, C1 and C2, specifically within the second flour stream, which is of coarser granulation, compared to the first flour stream from the same passage (Appendix A, Table A1). From these passages, usually two, or sometimes even three, different flour streams with distinct granulations are extracted. Similar occurrences were noted in the other two mills, especially in mill C, where many flour stream UCD values were negative (Appendix A, Table A2 and Table A3). However, it is notable that in instances where UCD values were negative or unrealistically low, the iodine absorption level (Ai%) values were comparable to those of streams from other grinding passages. Correlating two datasets revealed no significant difference in the correlation coefficient between UCD and Ai values, whether considering or excluding grinding passages with negative values. The correlation coefficient was approximately at the maximum value of 0.99. This suggests the practicality of expressing results in Ai values in situations where UCD values are rather unreliable.

Furthermore, a notable negative correlation coefficient of −0.88 was established between flour granulation, represented by the average particle size of passage flours, and starch damage expressed as Ai% (Table A1, Table A2 and Table A3, Appendix A). This negative correlation suggests that starch damage increases with the decreasing of flour granulation, which is in accordance with the expected assumption that higher comminution levels lead to elevated starch damage, and also with the results of the previous works investigating a similar topic [4,9,29].

**Figure A1:**
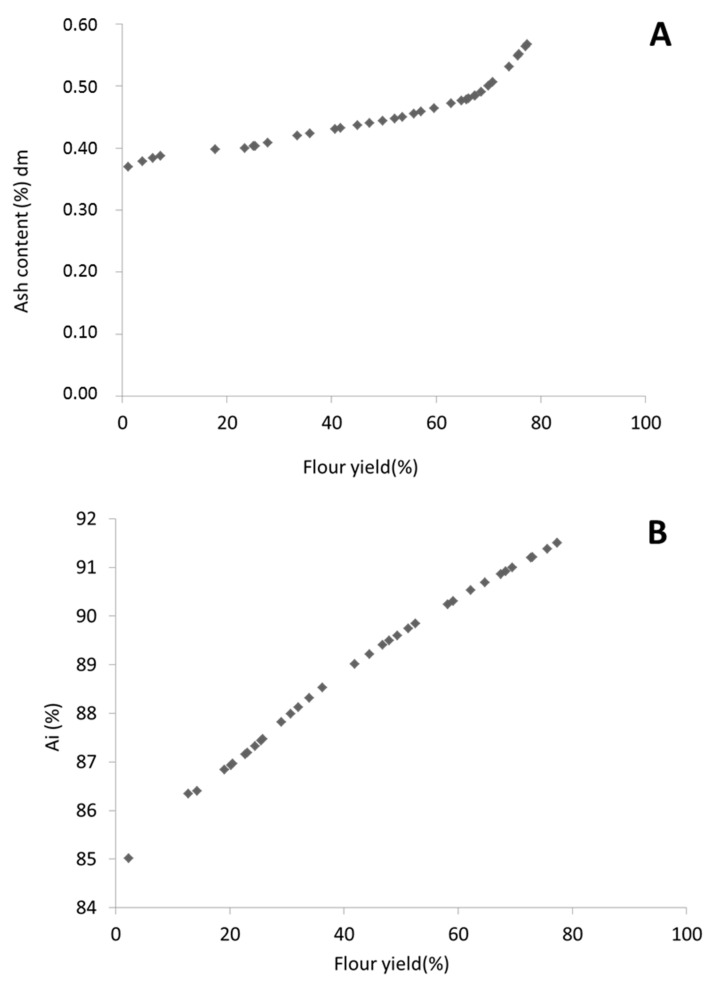
Cumulative-ash (**A**) and damaged-starch (**B**) curves for mill A.

**Figure A2:**
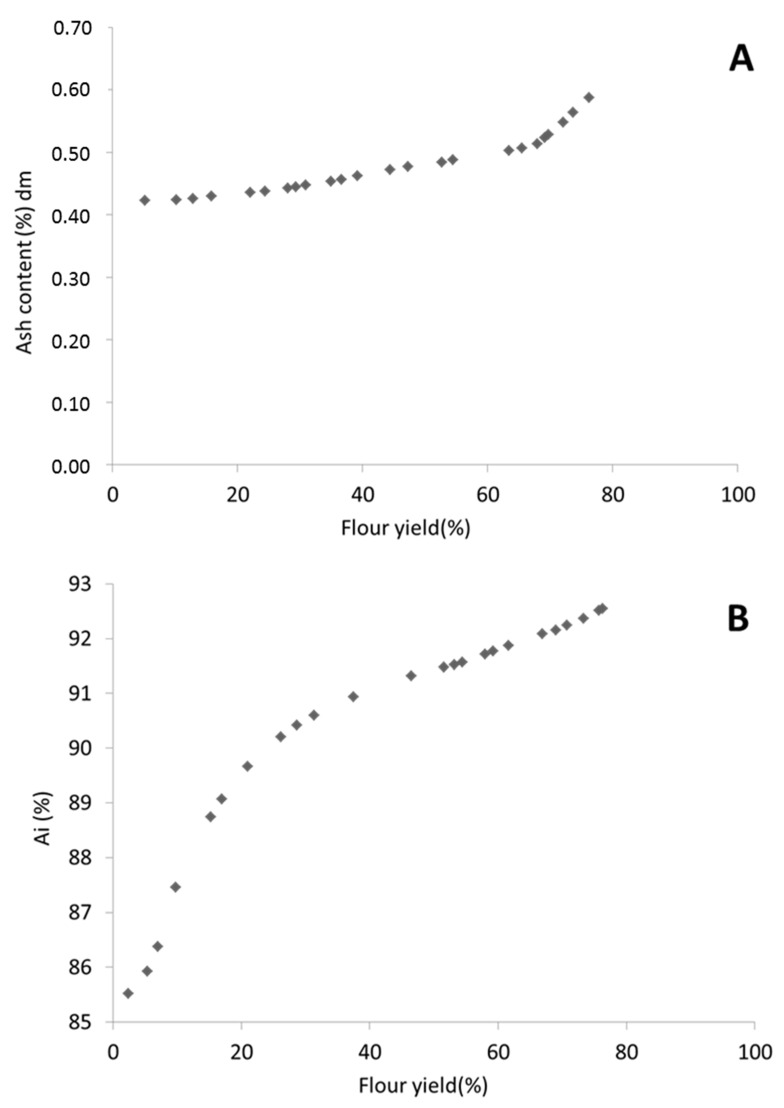
Cumulative-ash (**A**) and damaged-starch (**B**) curves for mill B.

**Figure A3:**
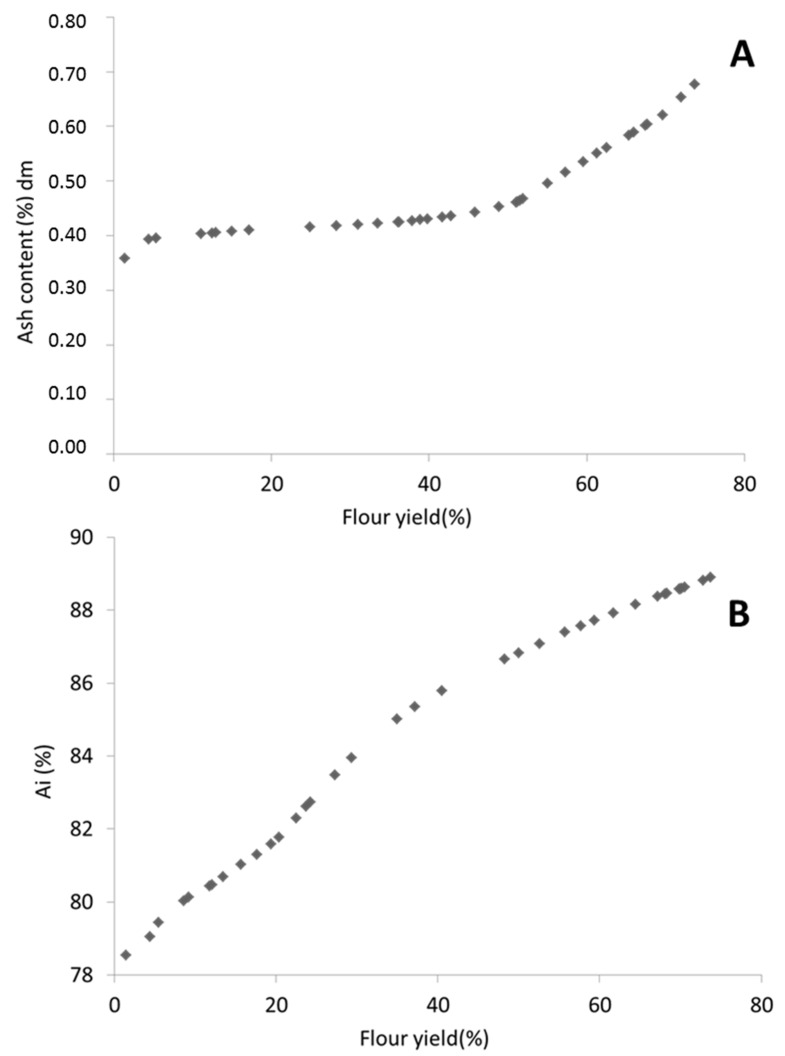
Cumulative-ash (**A**) and damaged-starch (**B**) curves for mill C.

Regarding the production of damaged starch observed across passages, damaged starch production mildly increased from front to back passages in the break system, and this trend was observed in all three mills. In the sizing and reduction system, starch damage intensifies from the initial to the final grinding passages, with the minimum damage occurring at passages C1, CL1, and CL2, and the maximum at passages C5 and C6. On the other hand, the flour yield from the first three passages in both the sizing and reduction system in total was 66.29% (Mill A), 53.97% (Mill B), and 47.77% (Mill C). Thus, it can be concluded that starch damage control is primarily achievable at the front passages of the sizing and reduction system, since these passages generate the majority of the flour release in the mill. All observed trends were in accordance with previous research investigating the current topic [13,22,30], and were also suggested by the cumulative curves for ash and damaged starch (Figure A1, Figure A2 and Figure A3, Appendix A).

The cumulative ash curve illustrates the relationship between cumulative flour ash content and cumulative flour yield. It is constructed based on the flow rate, percentage of ash, and moisture level of all intermediate flour streams in the mill. These streams are arranged in increasing order of ash content, starting with the lowest-ash flour. Calculations are performed by blending two streams at a time, followed by the addition of higher-ash-content flour to the blend until all streams are accounted for [31]. From a technical standpoint, the cumulative ash curve serves as a key indicator of mill efficiency [15], where optimal effectiveness is suggested by minimal curve increase in the area of low flour-extraction rate. In all three mills tested, the cumulative ash curve had a standard shape and did not significantly increase in the area of low flour extraction. Following the same principle used to form the cumulative ash curve, a cumulative curve for damaged starch was formed. Curves for damaged starch were almost linear in all three mills and, in the first half of the curves, a much more direct increase in starch damage compared to ash content was observed. The similarity between these two types of curves lies in the “second half” of the curves, which include flour streams from the end passages of the reduction system having higher ash content and greater starch damage; namely, as starch damage and ash content increases from the initial to the final passages in the break, sizing and reduction systems, higher flour extraction results, with an upward trend in both curves.

The difference suggested by the cumulative ash and damaged starch curves was also recognized by correlation coefficient between starch damage and ash content, which for all three mills was 0.50. However, comparative analysis of two datasets in Mill B (passages C4, C5, and C6) revealed an increased correlation coefficient of 0.83 between starch damage and ash content, indicating a relatively strong correlation. The research of Thiele [32] conducted at Kansas State University pilot mill also suggests that there is a connection between starch damage and ash content, particularly at the later stages of the reduction system (where damaged starch content in the flour is the highest, while ash content in the flour is higher compared to flours from initial passages of the reduction system), but that these two parameters do not influence one another. Same authors also suggested that narrowing the roll gap, in comparison to the usual roll gap, did not affect flour yield but did affect the increase in damaged starch. Moreover, the cumulative ash curves were very similar in the case of the standard and narrower roll gap. On the other hand, when the roll gap was set to be wider, flour yield decreased, along with flour ash content.

Since it is known that grinding parameters influence milling efficiency indicators, such as flour yield, damaged starch and energy consumption, and since it has been shown that starch damage control is primarily achievable at front passages of the sizing and reduction system, where the majority of the flour is obtained, in the second stage of the research, the influence of the grinding parameters on the efficiency of the reduction system of the wheat flour milling process was investigated.

### 3.2. Second Stage

A total of 17 experimental runs were determined by the Box–Behnken design. The recommended order of milling parameter combinations and the obtained responses, i.e., damaged starch parameters (Ai and UCD), flour yield and energy consumption are presented in Table 2.

Regression coefficients are represented in Table 3, where a star denotes input factors, their interactions and their quadratic terms, which expressed statistically significant influence on the observed responses, according to *p* values from the ANOVA table (*p* < 0.05). Coefficient of determination (R^2^) was used to check if the applied model provides proper representation of the experimental data. Lack of fit testing for damaged starch responses (R1 and R2) confirmed the adequacy of fitting the experimental data to a second-order polynomial model, since *p* values for lack of fit were insignificant (*p* > 0.05). On the other hand, the lack-of-fit coefficients for R3 and R4 responses were significant. However, *p* values for the model for all four responses were recognized as statistically significant (*p* < 0.05).

**Figure 1:**
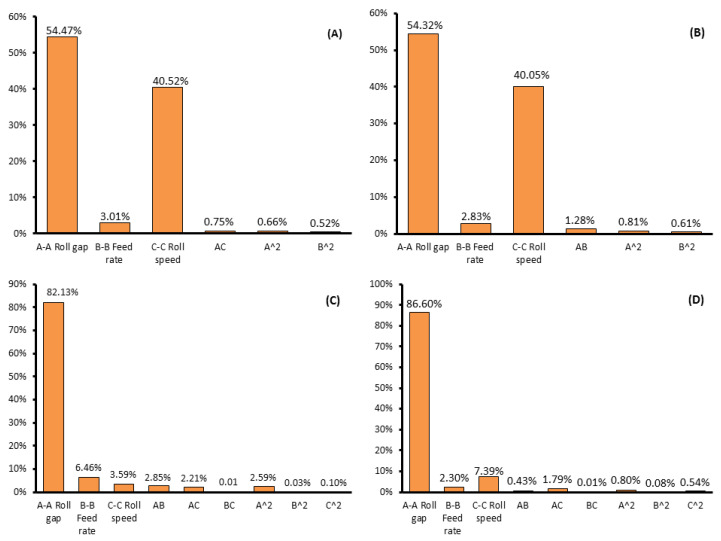
Contribution plot of milling parameters influencing (**A**) iodine absorption (Ai%); (**B**) UCD; (**C**) flour yield; and (**D**) energy consumption.

Contribution plots of the milling parameters influencing investigated responses are represented in Figure 1.

#### 3.2.1. Influence of Milling Parameters on the Damaged Starch

The damaged starch, as expressed in iodine absorption (Ai (%)) and UCD units ranged from 92.12 to 93.57, and from 15.9 to 19.8, respectively (Table 3). From Table 3 it can be noticed that same parameters (linear parameter of roll gap and roll speed) statistically significantly influenced observed responses, and in approximately the same contribution (Figure 1A,B). Also, R^2^ values were almost the same, while the lack-of-fit value was statistically insignificant in both cases (Table 3).

Generally, there is a direct correlation between flour fineness and starch damage, and finer flour should imply higher damaged starch content [33]. In that context, reducing the roll gap was anticipated to expose the material to stronger deformation forces, leading to increased particle size reduction, i.e., finer flour production, and, consequently, higher damaged starch content in obtained flour. Similarly, an increase in feed rate was expected to shorten the time each particle is exposed to deformation forces, resulting in lower particle size reduction and therefore damaged starch content. Furthermore, higher roll speeds were expected to increase particle deformation due to faster transmission of deformation forces from rolls to milled material, thus increasing particle size reduction and damaged starch content [14,18]. Although it has been demonstrated that milling parameters affect the particle size reduction, i.e., flour yield, in the expected manner (as shown in Table 3 and as further discussed in the next section), it was found that the milling parameters expressed an opposite trend for damaged starch content to that expected.

**Figure 2:**
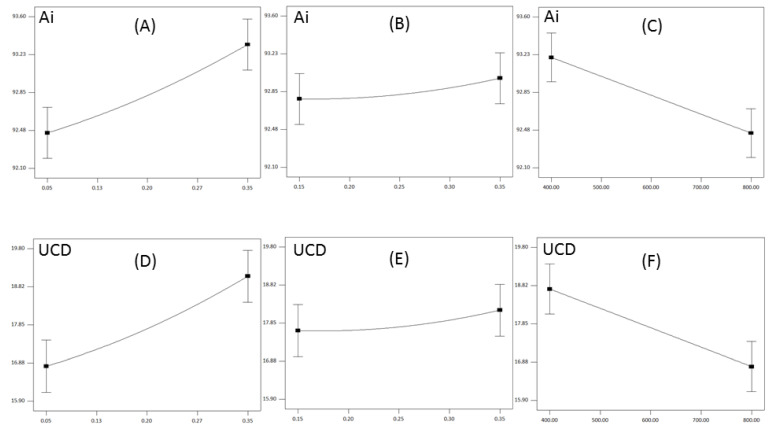
Influence of the roll gap (**A**,**D**), feed rate (**B**,**E**) and roll speed (**C**,**F**) on the Ai and UCD values.

As shown in Figure 2, with the decrease in the roll gap, the damaged starch content reduced (Figure 2A,D), while increasing the feed rate had a mildly positive effect on the observed response (Figure 2B,E). Also, higher roll speeds led to a decrease in damaged starch content in the flour (Figure 2C,F).

A potential reason for the unexpected trend could be the variability in the damaged starch content in the samples used for milling. As mentioned in the Section 2, the initial sample was fine semolina, of which the total mass was 50 kg. Those 50 kg were divided into 500 g samples and taken for milling. After milling, every sample was sieved to obtain flour, and then 1 g of each flour sample was taken for damaged starch analysis.

There is a possibility that the damaged starch content changed in line with the expected influence of the milling parameters, but that the variation in the initial damaged starch content may have influenced the trend of parameter effects only being noticeable on individual samples, rather than the entire set of 17 milled samples. More precisely, the results would potentially be more relevant if the relative increase in damaged starch for each of the 17 milled samples was observed, and then the relative increases for each sample were compared between samples. This approach could be applied in future research to further illuminate the issue of damaged starch generation in the wheat flour production process.

#### 3.2.2. Influence of Milling Parameters on the Flour Yield

The flour yield ranged between 0.5% and 27.61% (Table 3). The *p*-values from the ANOVA table indicated the significant impact of all linear terms of the selected milling parameters and the quadratic term of the roll gap on the observed response. Furthermore, the analysis suggests that the interactions between the roll gap and feed rate, as well as the roll gap and roll speed, had a significant influence on flour yield. Figure 1C illustrates the contribution plot of milling parameters affecting flour yield, and the high coefficient of determination (R^2^ = 0.9809) presented in Table 3 indicates that the proposed model adequately represents the observed experimental data.

**Figure 3:**
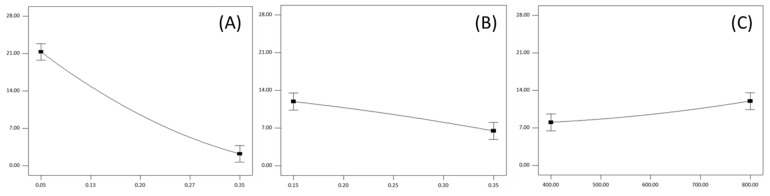
Influence of the roll gap (**A**), feed rate (**B**) and roll speed (**C**) on the flour yield.

A decrease in the roll gap resulted in a higher flour yield (Figure 3A). The observed trend aligns with established principles in roller milling, where a reduction in the roll gap corresponds to an increase in flour yield [16,17,34,35]. This phenomenon can be attributed to the dynamics of the milling process. When the roll gap is reduced, the particles are subjected to higher levels of stress and compression forces. Consequently, this intensified mechanical action leads to a greater number of fractures within the particles, facilitating a more efficient reduction in particle size. Also, a narrower roll gap, in consequence, increases the grinding zone, which causes prolonged grinding action.

In contrast, widening the roll gap diminishes the stress and compression forces exerted on the particles. This reduction in mechanical action results in fewer fractures and a less effective reduction in particle size which, in the end, results in a lower flour yield.

An increased feed rate reduced flour yield (Figure 3B). This trend is in accordance with ribbon theory [36], which suggests that increased feed rate reduces the amount of grinding any given particles receives, thus influencing negatively the flour yield. With an increased feed rate, a greater quantity of material requires milling within the same period of time. In this way, the elevated feed rate disperses the intensity of grinding action across a larger number of particles, which results in each individual particle being less exposed to deformation forces. Therefore, elevating feed rate leads to a lower flour yield. On the other hand, an increase in the roll speed had a positive impact on the flour release (Figure 3C), since the transmission of the deformation forces from rolls to particles of the grinded material is improved with higher roll velocities. The following trend was rather expected, and in accordance with previous studies [16,37].

The interaction of the roll gap, both with feed rate and with roll speed expressed a statistically significant impact on the flour yield. Consistent with the previously described trend for linear parameters, a higher flour yield was achievable when milling at lower roll gap values and feed rate, and at higher roll speeds. Thus, the highest flour yield (27.61%) was achieved in run 13, where the roll gap and specific load were set to a minimum, while the roll speed was 600 r/min. Conversely, employing identical conditions for roll gap and speed, but with the feed rate set to maximum (Run 16), resulted in nearly halving the flour yield (14.72%). When a larger amount of milling material is subjected to roll action, the intensity of the roll forces disperses across more particles. Consequently, each individual particle experiences reduced stress forces, which results in lesser particle-size reduction.

#### 3.2.3. Influence of Milling Parameters on the Energy Consumption

**Figure 4:**
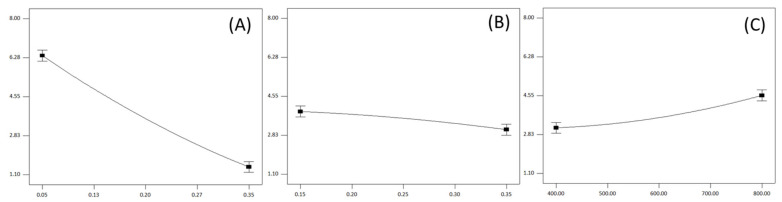
Influence of the roll gap (**A**), feed rate (**B**) and roll speed (**C**) on the energy consumption.

While each linear term exhibited statistically significant influence on the observed response (Table 3), it is noteworthy that the roll gap demonstrated the most dominant impact, accounting for approximately 87% in the sum of linear and quadratic terms related to energy consumption (Figure 1D). Widening the roll gap resulted in reduced energy consumption (Figure 4A). This can be explained by the fact that higher roll gap levels decrease the power required to overcome particle resistance during the milling process. Therefore, an increased roll gap had a negative impact on energy consumption. Conversely, increasing the roll velocity had a statistically positive impact on energy consumption (in practical terms this is a negative occurrence, since it means that energy consumption increased), as anticipated (Figure 4C). Maintaining elevated levels of roll velocities necessitates higher driving power, aligning with the expectation that these factors would statistically positively (negatively, from practical point of view) influence the response. Both of these trends are consistent with findings from previous studies researching the same topic [16,17,38].

While expected that higher feed rates would lead to an increase in energy consumption due to a greater mass flow of milled material, the trend of the influence was observed to be the opposite (Figure 3B). This contradiction in impact trends can be explained by Equation (1), which is used for calculating the observed response. In this equation, the mass of material (M) was constant (500 ± 0.01 g), making factors such as power during roll stand operation with material flow (Pw) and milling time (t) pivotal. Power values ranged from 0.95 to 2.3 kW, and an increase in feed rate led to higher power values, as expected, to overcome the elevated mass flow of material. Nevertheless, with the feed rate rising from 0.15 kg/cm/min to 0.35 kg/cm/min, milling time was significantly reduced for all observed runs, approximately from 20 s to 8 s, respectively. Consequently, the difference in milling time for runs with varying feed rates exerted a more pronounced influence on energy consumption than the power requirements of the roll stand during milling operations. Therefore, while an increase in feed rate necessitated higher power consumption, it exhibited a negative impact on the overall energy consumption (Figure 4B).

### 3.3. Implementation of Obtained Models in Production of Flour with Desired Damaged Starch Content, Yield, and Energy Consumption

In the final phase of the study, the potential implementation of the obtained models was considered, aiming to produce flour with an appropriate level of damaged starch, maximizing yield, and minimizing energy consumption. For this purpose, guidelines from the literature were utilized, specifically focusing on the optimal content of damaged starch in flour used for the production of various bakery products (Table 4).

Based on the obtained models, diverse requirements for flour can be specified. Given that the range of damaged starch was between 15.9 and 19.8, this interval was considered. Additionally, a technologically justified and rational demand would be to maximize the yield of such flour while minimizing energy consumption. Some of the requirements and proposed solutions, along with an assessment of meeting the specified demand, are presented in Table 5.

Various possibilities for the content of damaged starch in flour were considered, assigning different importance to the output parameters. Initially, the highest importance (5) was assigned to damaged starch, while the significance for flour yield and energy consumption was set to 2 and 1, respectively. The milling conditions to achieve these requirements are given in examples 1, 3, and 5.

It can be observed that the highest desirability was achieved for requirement 3, where UCD was set to be 18, which is the value that is close to the middle value of the observed response interval. Additionally, it is noticeable that in the case of the requirement for high damaged-starch content (requirement 1; UCD = 19), flour yield and energy consumption are predicted to be low. Conversely, when the requirement for the damaged starch content in the flour is for it to be low, there is an increase in the yield of such flour and energy consumption. In cases where similar requirements are set, but with an increased significance for flour yield and energy consumption at level 3 (requirements 2, 4, 6), desirability, as expected, decreases, while the trend of expected responses in that cases is identical to requirements 1, 3, and 5.

Finally, cases were observed where requirements were set not to target a specific value for the UCD of the obtained flour, but a certain interval (requirements 7 and 8). It was noticed that desirability in both cases decreased, compared to the previously observed cases. This is rather unexpected, since the assumption is that the model will more easily achieve flour with a damaged starch level within a certain interval than with a concrete value. However, there was an increase in flour yield in the case of such set requirements, with energy consumption having an approximately average value. It is also noticeable that in both cases, the model aimed for the UCD value to be closer to 18, allowing better desirability, as previously observed (requirement 3).

## 4. Conclusions

Starch damage increases progressively from the initial to the final passages during milling in the break, sizing and reduction systems. Appropriate monitoring of the degree of damaged starch is primarily based on controlling the following parameter during milling in the sizing system and the initial milling passages of the reduction system.

The study revealed that all investigated milling parameters significantly influenced flour yield and energy consumption, while for the damaged starch content a statistically significant influence was expressed for roll gap and roll speed.

Based on the obtained models, diverse requirements for flour were specified and various possibilities for the content of damaged starch in flour were considered. The models provided a set of milling runs for the production of different types of flours, suitable for various bakery products. Future research should focus on investigating the functionality of flours produced based on the models developed in the following study and the quality of different types of baked foods made from such obtained flours.

This analysis provides valuable insights into the milling process and its effects on flour quality. The findings contribute to a deeper understanding of starch damage during milling, and can inform strategies for optimizing milling operations to enhance flour quality regarding damaged starch content.

## Figures and Tables

**Table 1:** Complete overview of all three mills, with all grinding passages, flours, flour yield and starch damage values in the following passage flours.

**BREAK SYSTEM**										
	**Mill A**			**Mill B**			**Mill C**			
**Passage**	**Flour**	**Flour** **yield (%)**	**Ai (%)**	**Flour**	**Flour** **yield (%)**	**Ai (%)**	**Flour**	**Flour** **yield (%)**		**Ai (%)**
B1	1	2.80	88.96	1	7.61	91.05	1	7.67		90.55
	2	1.77	89.54							
B2	1	4.14	90.7	1	3.59	92.57	1	10.52		91.12
	2	2.99	92.88							
B3	1	3.31	92.57	1	12.47	92.92	1	1.28		93.48
	2	1.26	94.37							
B4	1	1.54	93.94	1	1.70	94.16	1	3.72		93.38
B5	1	1.05	95.14		-	-	1	1.22		95.22
**SIZING SYSTEM**										
	**Mill A**			**Mill B**			**Mill C**			
**Passage**	**Flour**	**Flour** **yield (%)**	**Ai (%)**	**Flour**	**Flour** **yield (%)**	**Ai (%)**	**Flour**	**Flour** **yield (%)**	**Ai (%)**	
CL 1	1	3.13	94.73	1	4.97	94.07	1	3.74	93.43	
	2	2.46	91.54	2	2.21	87.88	2	1.34	85.46	
	3	1.87	86.92	3	1.83	93.09	3	0.88	81.47	
CL 2	1	3.63	94.88	1	5.60	92.21	1	3.42	92.24	
	2	13.53	86.64	2	3.41	85.515	2	1.89	82.68	
	3	1.43	88.47	3	2.33	92	3	1.93	78.55	
CL 3	1	3.31	96.17	1	2.99	94.54	1	4.53	90.69	
	2	2.95	91.66				2	2.59	83.51	
CL 4	-	-	-	1	2.40	95.615	1	2.78	90.33	
	-	-	-				2	2.36	84.37	
**REDUCTION SYSTEM**										
	**Mill A**			**Mill B**			**Mill C**			
**Passage**	**Flour**	**Flour** **yield (%)**	**Ai (%)**	**Flour**	**Flour** **yield (%)**	**Ai (%)**	**Flour**	**Flour** **yield (%)**	**Ai (%)**	
C 1	1	7.29	92.18	1	3.64	92.61	1	4.14	89.3	
	2	6.25	88.14	2	4.10	86.275	2	4.19	81.08	
	3	0.46	88.73	3	7.21	92.4	3	4.07	79.29	
C 2	1	7.29	93.97	1	8.62	92.635	1	2.97	90.69	
	2	2.17	90.87				2	3.00	83.10	
	3	3.02	85.02				3	3.50	81.48	
C 3	1	4.22	95.62	1	7.07	92.93	1	2.66	92.66	
	2	1.84	93.1				2	2.92	87.26	
	3	1.41	89.91				-	-		
C 4	1	2.49	93.65	1	7.27	94.53	1	2.28	92.73	
	2	0.46	89.43				2	0.52	94.60	
C 5	1	4.04	94.66	1	3.31	96.775	1	2.05	93.61	
	2	0.29	96.01				2	0.18	93.64	
C 6	1	1.59	95.42	1	0.79	97.185	1	2.42	91.38	
	2	1.74	91.16				2	0.23	94.01	
C 7	1	1.56	92.98	-	-	-	-	-	-	
	2	0.39	90.28							
**BREAK GRADER**										
	**Mill A**			**Mill B**			**Mill C**			
**Passage**	**Flour**	**Flour** **yield (%)**	**Ai (%)**	**Flour**	**Flour** **yield (%)**	**Ai (%)**	**Flour**	**Flour** **yield (%)**	**Ai (%)**	
P 1	-	-	-	-	-	-	1	1.68	88.51	
	-	-	-	-	-	-	2	1.45	81.03	
P 2	-	-	-	-	-	-	1	0.68	88.61	
	-	-	-				2	0.39	81.89	
**TAILINGS SYSTEM**										
	**Mill A**			**Mill B**			**Mill C**			
**Passage**	**Flour**	**Flour** **yield (%)**	**Ai (%)**	**Flour**	**Flour yield (%)**	**Ai (%)**	**Flour**	**Flour** **yield (%)**	**Ai (%)**	
T 1	-	-	-	-	-	-	1	3.09	94.72	
**VIBRO SIFTER**										
	**Mill A**			**Mill B**			**Mill C**			
**Passage**	**Flour**	**Flour** **yield (%)**	**Ai (%)**	**Flour**	**Flour** **yield (%)**	**Ai (%)**	**Flour**	**Flour** **yield (%)**	**Ai (%)**	
V 1	1	0.00	-	1	3.31	94.495	1	3.15	93.23	
V 2	2	0.04	96.83	2	3.58	95.965	2	4.30	92.59	
V 3	3	2.26	96.42	-	-	-	3	0.27	93.6	

**Table 2:** The Box–Behnken experimental design and obtained responses.

Run	Roll Gap [mm]	Feed Rate [kg/cm min]	Roll Speed [n/min]	Ai (%)	UCD	Flour Yield (%)	Energy Consumption [wh/kg]
1	0.2	0.25	600	92.22	16.2	9.44	3.67
2	0.35	0.25	800	92.83	17.8	2.53	1.66
3	0.2	0.15	800	92.39	16.6	13.3	5
4	0.05	0.25	800	92.22	16.2	25.59	8
5	0.2	0.15	400	93.16	18.7	8.82	3.33
6	0.2	0.35	800	92.67	17.4	10.44	4.01
7	0.2	0.25	600	92.89	17.9	9.81	3.66
8	0.05	0.25	400	93.13	18.6	17.62	5.66
9	0.2	0.25	600	92.89	17.9	8.68	3.53
10	0.35	0.35	600	93.56	19.7	0.5	1.41
11	0.2	0.35	400	93.38	19.2	6.23	2.59
12	0.2	0.25	600	92.92	18	9.12	3.46
13	0.05	0.15	600	92.12	15.9	27.61	6.67
14	0.35	0.25	400	93.45	19.4	3.34	1.33
15	0.35	0.15	600	93.57	19.8	3.41	1.66
16	0.05	0.35	600	92.45	16.8	14.72	5.43
17	0.2	0.25	600	93.1	18.5	10.45	3.53

**Table 3:** Regression equation coefficients for responses.

	Responses			
	R1	R2	R3	R4
Intercept				
$\beta_{0}$	93.8869	19.08344	28.45392361	6.3847
Linear				
$\beta_{1}$	0.2794 *	8.39474 *	−101.9944444 *	−16.3080 *
$\beta_{2}$	−1.9158	−2.46272	−44.99166667 *	−0.2037 *
$\beta_{3}$	−0.0024 *	−0.0049375 *	0.011770833 *	−0.0003 *
Interaction				
$\beta_{12}$		−16.66667	166.3333333 *	16.4352
$\beta_{13}$	0.0024		−0.073166667 *	−0.0167 *
$\beta_{23}$			−0.003375	−0.0031
Quadratic				
$\beta_{11}$	2.9474	8.59649	102.9444444 *	14.49691358 *
$\beta_{22}$	5.8816	16.84211	−25.625	−10.4375
$\beta_{33}$			0.00001134	0.00000667
Lack of fit	0.7595	0.7344	0.0204	0.0116
$R^{2}$	0.7711	0.7721	0.9809	0.9929

* Statistically significant at *p* < 0.05.

**Table 4:** Approximate levels of damaged starch (UCD) to produce specific types of baked products (from KPM analytics [12]).

Product	Cookies	Noodles	Crackers	Hearth Bread	Flat Bread	Tortillas	Pan Bread
UCD range (approximately) *	13.7–16.4	15–17.5	15.8–18.5	15.8–20	17–20.5	19–21	19–23

* range is provided on 12% level-protein basis.

**Table 5:** Criteria for flour with milling parameters, responses and desirability for all examples.

Request				Solution						
No.	Response	Goal	Importance	A	B	C	UCD	Yield	Energy	Desirability
1	UCD	target = 19.5	5	0.29	0.15	400	19.50	5.58	2.21	0.66
	Yield	maximize	2							
	Energy	minimize	1							
2	UCD	target = 19.5	5	0.26	0.15	400	19.20	7.00	2.56	0.63
	Yield	maximize	3							
	Energy	minimize	3							
3	UCD	target = 18	5	0.13	0.15	400	18.00	15.68	4.52	0.80
	Yield	maximize	2							
	Energy	minimize	1							
4	UCD	target = 18	5	0.13	0.15	400	18.00	15.68	4.52	0.72
	Yield	maximize	3							
	Energy	minimize	3							
5	UCD	target = 16	5	0.12	0.15	800	16.00	22.27	6.64	0.78
	Yield	maximize	2							
	Energy	minimize	1							
6	UCD	target = 16	5	0.16	0.15	800	16.34	18.01	5.71	0.64
	Yield	maximize	3							
	Energy	minimize	3							
7	UCD	in range from 16 to 18	5	0.05	0.15	400	17.47	21.89	5.80	0.59
	Yield	maximize	2							
	Energy	minimize	1							
8	UCD	in range from 18 to 19.8	5	0.13	0.15	400	18.00	15.68	4.52	0.55
	Yield	maximize	2							
	Energy	minimize	1							

## Data Availability

The original contributions presented in the study are included in the article, further inquiries can be directed to the corresponding author.

## Appendix A

**Table A1:** Mill A passage analysis results.

BREAK SYSTEM								
Passage	Flour	Flour Yield (%)	D [0.5] [µm]	D [4.3] [µm]	Ai (%)	AACC (%)	UCD	Ash (%)dm *
B1	1	2.80	85.50	167.03	88.96	2.88	7.05	0.57
	2	1.77	76.10	122.80	89.54	3.11	8.75	0.59
B2	1	4.14	64.30	94.70	90.7	3.78	12.65	0.50
	2	2.99	67.40	141.30	92.88	4.77	16.80	0.53
B3	1	3.31	55.40	65.30	92.57	5.27	18.60	0.51
	2	1.26	55.21	66.80	94.37	5.99	20.95	0.63
B4	1	1.54	58.10	65.10	93.94	5.99	20.90	0.85
B5	1	1.05	52.30	61.20	95.14	7.40	24.90	1.07
**SIZING SYSTEM**								
**Passage**	**Flour**	**Flour Yield** **(%)**	**D [0.5]** **[µm]**	**D [4.3]** **[µm]**	**Ai (%)**	**AACC** **(%)**	**UCD**	**Ash** **(%)dm**
CL 1	1	3.13	45.80	56.80	94.73	6.48	22.40	0.46
	2	2.46	87.90	96.50	91.54	4.20	14.55	0.40
	3	1.87	130.40	135.80	86.92	2.46	1.95	0.40
CL 2	1	3.63	54.60	139.70	94.88	6.73	23.10	0.38
	2	13.53	153.00	161.40	86.64	2.43	0.75	0.41
	3	1.43	124.10	129.10	88.47	2.86	6.85	0.37
CL 3	1	3.31	66.30	278.10	96.17	8.15	26.80	0.59
	2	2.95	123.60	133.70	91.66	4.43	15.50	0.50
**REDUCTION SYSTEM**								
**Passage**	**Flour**	**Flour Yield** **(%)**	**D [0.5]** **[µm]**	**D [4.3]** **[µm]**	**Ai (%)**	**AACC** **(%)**	**UCD**	**Ash** **(%)dm**
C 1	1	7.29	80.50	90.70	92.18	4.57	16.10	0.47
	2	6.25	129.10	137.20	88.14	2.78	5.85	0.49
	3	0.46	125.90	132.60	88.73	2.90	7.20	0.46
C 2	1	7.29	68.50	138.10	93.97	5.89	20.65	0.41
	2	2.17	111.00	114.50	90.87	4.03	13.75	0.44
	3	3.02	159.70	162.90	85.02	2.44	-3.00	0.48
C 3	1	4.22	38.10	51.20	95.62	7.89	26.10	0.61
	2	1.84	79.20	85.60	93.1	5.55	19.55	0.55
	3	1.41	118.90	123.00	89.91	3.35	10.35	0.49
C 4	1	2.49	88.20	94.40	93.65	5.64	19.90	0.62
	2	0.46	130.50	134.30	89.43	3.07	8.50	0.68
C 5	1	4.04	67.80	78.60	94.66	6.55	22.60	1.10
	2	0.29	76.60	132.30	96.01	7.94	26.25	1.32
C 6	1	1.59	66.70	72.00	95.42	7.14	24.25	0.74
	2	1.74	134.80	141.30	91.16	3.97	13.55	0.96
C 7	1	1.56	138.20	145.10	92.98	5.10	18.05	1.36
	2	0.39	155.70	163.30	90.28	3.58	11.60	1.46
**VIBRO SIFTER**								
**Passage**	**Flour**	**Flour Yield** **(%)**	**D [0.5]** **[µm]**	**D [4.3]** **[µm]**	**Ai (%)**	**AACC** **(%)**	**UCD**	**Ash** **(%)dm**
V 2	2	0.04	14.30	20.35	96.83	8.30	27.15	0.79
V 3	3	2.26	28.30	42.80	96.42	8.08	26.60	1.29

* ash content provided on dry-matter basis.

**Table A2:** Mill B passage analysis results.

BREAK SYSTEM								
Passage	Flour	Flour Yield (%)	D [0.5] [µm]	D [4.3] [µm]	Ai (%)	AACC (%)	UCD	Ash (%)dm *
B1	1	7.61	39.90	84.80	91.06	3.87	13.05	0.55
B2	1	3.59	53.00	67.00	92.57	4.83	17.10	0.54
B3	1	12.47	66.30	79.20	92.93	5.11	18.05	0.59
B4	1	1.70	61.20	72.90	94.16	5.50	21.35	1.05
**SIZING SYSTEM**								
**Passage**	**Flour**	**Flour Yield** **(%)**	**D [0.5]** **[µm]**	**D [4.3]** **[µm]**	**Ai** **(%)**	**AACC** **(%)**	**UCD**	**Ash** **(%)dm**
CL 1	1	4.97	67.30	82.40	94.07	6.04	21.10	0.48
	2	2.21	134.70	141.80	87.88	2.65	4.55	0.49
	3	1.83	73.60	88.60	93.09	6.61	18.50	0.49
CL 2	1	5.60	95.20	106.10	92.21	4.58	16.10	0.50
	2	3.41	152.80	158.70	85.52	2.44	−1.70	0.46
	3	2.33	130.30	336.80	92.00	4.44	15.60	0.52
CL 3	1	2.99	64.20	77.30	94.54	6.46	22.35	0.61
CL 4	1	2.40	60.40	67.40	95.62	7.52	25.20	0.59
**REDUCTION SYSTEM**								
**Passage**	**Flour**	**Flour Yield** **(%)**	**D [0.5]** **[µm]**	**D [4.3]** **[µm]**	**Ai** **(%)**	**AACC** **(%)**	**UCD**	**Ash** **(%)dm**
C 1	1	3.64	96.20	102.00	92.61	4.86	17.20	0.43
	2	4.10	158.00	164.40	86.28	2.41	0.40	0.45
	3	7.21	87.70	94.60	92.40	5.59	16.65	0.42
C 2	1	8.62	85.30	90.90	92.64	4.99	17.25	0.45
C 3	1	7.07	83.50	87.80	92.93	7.23	18.05	0.42
C 4	1	7.27	73.60	82.00	94.53	6.45	22.30	0.55
C 5	1	3.31	53.10	60.40	96.78	8.80	28.30	0.73
C 6	1	0.79	63.30	71.10	97.19	8.40	29.40	1.12
**VIBRO SIFTER**								
**Passage**	**Flour**	**Flour Yield** **(%)**	**D [0.5]** **[µm]**	**D [4.3]** **[µm]**	**Ai** **(%)**	**AACC** **(%)**	**UCD**	**Ash** **(%)dm**
V 1	1	3.31	37.10	51.10	94.50	5.90	22.25	1.13
V 2	2	3.58	40.60	53.80	95.97	6.84	26.10	1.28

* ash content provided on dry-matter basis.

**Table A3:** Mill C passage analysis results.

BREAK SYSTEM								
Passage	Flour	Flour Yield (%)	D [0.5] [µm]	D [4.3] [µm]	Ai (%)	AACC (%)	UCD	Ash (%)dm *
B1	1	7.67	77.9	135.9	90.55	3.32	11.7	0.41
B2	1	10.52	70.3	82.1	91.125	3.60	13.25	0.43
B3	1	1.28	52.6	62.3	93.48	5.92	19.5	0.41
B4	1	3.72	73.6	95.7	93.385	5.46	19.25	0.45
B5	1	1.22	39.7	52.7	95.22	7.11	24.15	0.49
**SIZING SYSTEM**								
**Passage**	**Flour**	**Flour Yield** **(%)**	**D [0.5]** **[µm]**	**D [4.3]** **[µm]**	**Ai** **(%)**	**AACC** **(%)**	**UCD**	**Ash** **(%)dm**
CL 1	1	3.74	75.6	212.6	93.435	5.50	19.4	1.11
	2	1.34	143.7	149.3	85.46	2.44	−1.85	0.48
	3	0.88	189.4	289.1	81.47	3.44	−12.45	1.11
CL 2	1	3.42	82	132.2	92.24	4.61	16.25	0.45
	2	1.89	170.9	197.2	82.685	2.94	−9.15	0.42
	3	1.93	191.3	195.7	78.55	5.22	−20.25	0.36
CL 3	1	4.53	81.8	90.8	90.695	3.70	12.1	0.44
	2	2.59	153.3	158.3	83.51	2.69	−6.95	0.49
CL 4	1	2.78	90.9	96.7	90.33	3.51	11.1	0.42
	2	2.36	148.6	154.2	84.375	2.52	−4.65	1.66
**REDUCTION SYSTEM**								
**Passage**	**Flour**	**Flour Yield** **(%)**	**D [0.5]** **[µm]**	**D [4.3]** **[µm]**	**Ai** **(%)**	**AACC** **(%)**	**UCD**	**Ash** **(%)dm**
C 1	1	4.14	102.7	128.1	89.3	3.05	8.35	0.41
	2	4.19	162.9	168.9	81.08	3.98	−13.5	0.54
	3	4.07	177.6	183.9	79.295	4.70	−18.2	0.60
C 2	1	2.97	93.1	139.6	90.69	3.67	12.05	0.64
	2	3.00	155.3	160.7	83.105	2.80	−8.1	1.03
	3	3.50	172.4	177.8	81.485	3.41	1	0.45
C 3	1	2.66	83.1	91.9	92.665	4.90	17.35	1.21
	2	2.92	143.4	148.3	87.26	2.51	2.9	0.43
C 4	1	2.28	100.6	296.5	92.735	4.96	17.5	0.48
	2	0.52	34.3	85.7	94.605	6.52	22.5	0.96
C 5	1	2.05	89.6	342.12	93.615	5.65	19.85	1.16
	2	0.18	63.73	77.1	93.64	5.67	19.95	0.46
C 6	1	2.42	101.9	107.5	91.38	4.05	13.9	1.05
	2	0.23	58.7	69.7	94.01	5.98	20.9	0.67
**BREAK GRADER**								
**Passage**	**Flour**	**Flour Yield** **(%)**	**D [0.5]** **[µm]**	**D [4.3]** **[µm]**	**Ai** **(%)**	**AACC** **(%)**	**UCD**	**Ash** **(%)dm**
P 1	1	1.68	111.3	117.4	88.51	2.81	6.25	1.07
	2	1.45	162.7	169.5	81.03	3.63	−13.6	0.53
P 2	1	0.68	118.2	123.6	88.61	2.83	6.55	0.42
	2	0.39	162.8	167.9	81.895	3.23	−11.3	0.87
**TAILINGS SYSTEM**								
**Passage**	**Flour**	**Flour Yield** **(%)**	**D [0.5]** **[µm]**	**D [4.3]** **[µm]**	**Ai** **(%)**	**AACC** **(%)**	**UCD**	**Ash** **(%)dm**
T 1	1	3.09	62.1	93.9	94.72	6.63	22.85	1.02
**VIBRO SIFTER**								
**Passage**	**Flour**	**Flour Yield** **(%)**	**D [0.5]** **[µm]**	**D [4.3]** **[µm]**	**Ai** **(%)**	**AACC** **(%)**	**UCD**	**Ash** **(%)dm**
V 1	1	3.15	52.1	76.2	93.23	5.33	18.85	1.64
V 2	2	4.30	89.9	102.7	92.59	4.85	17.15	0.96
V 3	3	0.27	28.2	106.6	93.6	5.66	19.85	1.16

* ash content provided on dry-matter basis.
